# Family lifestyle dynamics and childhood obesity: evidence from the millennium cohort study

**DOI:** 10.1186/s12889-018-5398-5

**Published:** 2018-04-16

**Authors:** Laura A. Gray, Monica Hernandez Alava, Michael P. Kelly, Michael J. Campbell

**Affiliations:** 1 Health Economics and Decision Science, School of Health and Related Research, University of Sheffield, Sheffield, S1 4DA UK; 2 Institute of Public Health, University of Cambridge, Cambridge, CB2 0SR UK; 3 Design, Trials and Statistics, School of Health and Related Research, University of Sheffield, Sheffield, S1 4DA UK

**Keywords:** Childhood obesity, Family lifestyle, Dynamic latent factor model

## Abstract

**Background:**

The prevalence of childhood obesity has been increasing but the causes are not fully understood. Recent public health interventions and guidance aiming to reduce childhood obesity have focused on the whole family, as opposed to just the child but there remains a lack of empirical evidence examining this relationship.

**Methods:**

Using data from the longitudinal Millennium Cohort Study (MCS), we investigate the dynamic relationship between underlying family lifestyle and childhood obesity during early childhood. The MCS interviewed parents shortly after the birth of their child and follow up interviews were carried out when the child was 3, 5 and 7 years. We use a dynamic latent factor model, an approach that allows us to identify family lifestyle, its evolution over time (in this case between birth and 7 years) and its influence on childhood obesity and other observable outcomes.

**Results:**

We find that family lifestyle is persistent, 87.43% of families which were above the 95th percentile on the lifestyle distribution, remained above the 95th percentile when the child was 7 years old. Family lifestyle has a significant influence on all outcomes in the study, including diet, exercise and parental weight status; family lifestyle accounts for 11.3% of the variation in child weight by age 7 years.

**Conclusion:**

The analysis suggests that interventions should therefore be prolonged and persuasive and target the underlying lifestyle of a family as early as possible during childhood in order to have the greatest cumulative influence. Our results suggest that children from advantaged backgrounds are more likely to be exposed to healthier lifestyles and that this leads to inequalities in the prevalence of obesity. To reduce inequalities in childhood obesity, policy makers should target disadvantaged families and design interventions specifically for these families.

## Background

The prevalence of childhood obesity has been increasing; figures from the Health Survey for England (HSE) suggests that the prevalence of childhood obesity rose steadily between 1995 and 2004 before levelling off between 2004 and 2012 [1]. The prevalence of childhood obesity remains high and the causes of childhood obesity are not fully understood. Recent public health interventions and guidance which aim to reduce childhood obesity have focused on the whole family, as opposed to just the child, for example Change4Life [2] and clinical and public health guidelines from the National Institute for Health and Care Excellence [3–5]. In doing so, policy makers acknowledged an association between the way families live (what is often loosely called lifestyle) and childhood obesity. However, there is a lack of empirical evidence on this relationship.

Previous studies have shown strong relationships between the BMI of family members [6–9]. There are studies which claim that these correlations are due largely to genetic influences [10–14]. These studies are based on adoption or twin studies (a very specific part of the population) and generally look only at descriptive statistics and correlations, rather than accounting for other confounding factors. In studies which use more flexible and complex statistical techniques to account for a wider range of confounding factors generally suggest that this correlation is at least equally due to non-genetic influences, such as lifestyle or behavioural influences [8, 9, 15–20]. Correlations between spouses which are less likely to be a result of genetic influences than correlations between blood relatives, provide further support for the argument that shared lifestyle significantly influences correlations between family members [6, 8]. However, assortative mating could play a role here [21], meaning that resemblance in BMI between spouses is not entirely attributable to the shared environment or lifestyle. Many influences which might affect the likelihood of obesity in parents and children are considered to be unobservable [8]. Some studies lack the ability to identify the effects of environmental factors and as a result these effects are often underestimated and genetics are assumed to be the driving influence [19]. Abrevaya and Tang describe in detail the endogeneity caused by omitted variables when using one family member’s obesity to predict another’s. When attempting to measure this they found that education among other things was a source of endogeneity, however they could not measure the endogeneity caused by unobservable characteristics, not available in their data [6]; that is, there could be unobserved variables outside their analysis which is affecting the obesity status of both family members.

Childhood obesity has been shown to be significantly correlated with other observable behaviours, including hours spent watching television [22], diet and exercise [23, 24] and breastfeeding [25], amongst others. Better understanding the complex relationship between childhood obesity and other observable lifestyle indicators within the family could help to improve future interventions. Many studies use these behaviours as independent variables to predict childhood obesity, but again there is likely to be an underlying endogenous influence affecting all of these observable characteristics. Despite many studies showing that interventions have been successful in improving the nutrition or physical activity of children, relatively few studies have found a significant effect of these interventions on childhood adiposity [26]. ‘Life worlds’ [27] are intrinsic to understanding the development of childhood obesity. Life worlds refers to the way that an individual or family lives their life, the world directly experienced in the subjectivity of everyday life [28]. However, life worlds are difficult to study by virtue of their complexity, longevity and the problems attached to observing them. As a proxy for this, we have operationalised in this study what we call ‘underlying family lifestyle’. Changes to underlying family lifestyle might lead to benefits that can be identified across many of the observable outcomes. It is this underlying family lifestyle, which is the source of correlation across the observable outcomes. Through socialisation the way a family lives will impact on the child [29]. For this reason, there has been a consensus that family-based interventions should be used [30–33] and interventions which are targeted at all family members or parents only rather than child only interventions tend to be more effective [30, 31], particularly when aiming to prevent rather than treat childhood obesity. They can also be more cost-effective, since they can reduce obesity in multiple family members [34]. That is not to suggest that all family based interventions will be successful and some family-based interventions were found to be no better than child only interventions [35]. This emphasises the need for further research into the type of family-based intervention that are more likely to be successful.

Obesity is a very persistent trait [36], however, similar to the endogeneity described above, it is difficult to determine whether past obesity influences current obesity or whether a persistent underlying and unobservable factor is influencing obesity at all times. Socioeconomic status [37], parental education [38] and single-parenthood [39] have all been shown to influence obesity and are relatively consistent over time. However, it remains unclear what mechanisms are behind these relationships. From a policy perspective, if obesity were determined purely by past obesity and social circumstance, interventions to reduce obesity would be ineffective. However, it has been shown that interventions can be effective in reducing childhood obesity [40]. Similarly, interventions have been successful in reducing weight gain during pregnancy [41] and in reducing obesity in adults [42]. This suggests that with the right interventions obesity in both children and adults can be reduced, and that obesity is not solely determined by past obesity and social circumstance but by more complex interactions going on in family life.

Given that childhood obesity and other outcomes of family lifestyle are expected to be dependent on the same underlying influences, it is important to model these outcomes jointly. Despite this, the majority of previous studies have estimated these variables independently [43–45]. This approach is less informative when considering policy implications because it is only possible to identify how potential lifestyle interventions might influence a single outcome. Other studies have jointly estimated a range of observable lifestyle outcomes, including diet, alcohol consumption and smoking habits [46, 47], allowing the benefits of potential interventions to a range of outcomes to be investigated but have been unable to identify the underlying cause of the correlation between these variables.

Existing studies show that early-life influences of obesity, particularly lifestyle during pregnancy and early infancy are important in predicting later obesity [25, 48–50]. However, these studies are generally cross-sectional and do not allow the evolution of lifestyle behaviours over time to be investigated. These early-life influences might continue to have an effect throughout childhood and new influences could emerge as children grow up and their immediate environment changes, for example starting school. The use of more flexible dynamics when modelling development during childhood is encouraged because children change so rapidly [51].

We contribute to exiting literature by using a structural model to investigate how family lifestyle evolves over time during early childhood and how family lifestyle dynamics influence childhood obesity. This approach has a number of advantages. First, structural models can explain much more than models which use a single equation and can be used to investigate multiple and more ambitious research questions than more modest models such as fixed effects or instrumental variable models [52]. Second, unlike more commonly used autoregressive models, structural models allow parameter estimates to differ over time. Third, different mean outcomes can be identified for children with different characteristics unlike existing studies into adiposity which are restricted to estimating a single average treatment effect for a sample [53]. Identifying the full distribution of treatment effects allows those who will benefit most from potential interventions to be identified. This, coupled with the dynamic nature of the model, is vital evidence for policy makers in order for them to have the greatest possible impact.

## Methods

In order to investigate the dynamic influence that underlying family lifestyle has on our outcome of interest, childhood obesity, we use a dynamic latent factor model, similar to that used in previous studies [51, 54]. They use this approach to identify the formation of skills during early childhood, whilst we use it to explore the evolution of family lifestyle and its relationship with obesity. The model is made up a set of latent factors (sometimes known as measurement models) which identify the underlying lifestyle of a family using a range of outcomes and a structural model which estimates the relationship between these latent factors, in this case, the dynamic process of how family lifestyle evolves over time. Both parts of the model are outlined below and are jointly estimated using maximum likelihood. A more detailed explanation relating to structural models can be found in the literature [55, 56].

### Latent factor for family lifestyle

We are interested in the influence of underlying family lifestyle on childhood adiposity, so that

1 $$ {\boldsymbol{Y}}_{it}={\boldsymbol{\lambda}}_t{\boldsymbol{\theta}}_{it}+{\boldsymbol{\delta}}_t{\boldsymbol{W}}_{it}+{\boldsymbol{\xi}}_{it} $$

where ***Y***_*it*_ is the childhood adiposity outcome at time *t* of child *i*, ***θ***_*it*_ is underlying family lifestyle with corresponding factor loading ***λ***_*t*_ at time *t*, ***W***_*it*_ is a vector of independent variables influencing the adiposity outcome at time *t* with vector of coefficients ***δ***_*t*_ and ***ξ***_*it*_ is normally distributed error term. Previous adiposity is not included in this equation, as we assume any persistence in obesity is caused by a persistence in underlying lifestyle.

This underlying family lifestyle is unobservable and cannot be identified using this single equation. Due to the unobservable nature of this underlying family lifestyle, a latent factor is the only way to directly estimate it, allowing this underlying concept to be identified without measurement error [57]. There is multicollinearity between each of the estimated outcomes due to their shared dependence on underlying family lifestyle but by using a latent factor, this multicollinearity is accounted for. Multiple lifestyle outcomes have previously been jointly estimated using a multivariate probit model [46] allowing the correlation of the error terms in each of the outcome equations to be accounted. However, using this model, it is not possible to estimate directly the underlying factor that is influencing each of these observable outcomes and therefore it is not possible to estimate the effect that this underlying factor has on each outcome. This study directly estimates the underlying source of this correlation allowing its influence on each of the outcomes to be examined.

Similar to Eq. 1, each outcome depends on family lifestyle and is related to the underlying latent factor so that, for continuous outcome *k*.

2 $$ {\boldsymbol{Y}}_{kit}={\boldsymbol{\lambda}}_{kt}{\boldsymbol{\theta}}_{it}+{\boldsymbol{\xi}}_{kit}; $$

the error terms are equivalent to that in Eq. 1 and are independently and identically distributed. Other parameters are also equivalent to those in Eq. 1. In both Eqs. 1 and 2, continuous outcomes are estimated using a linear regression and discrete outcomes are estimated using probit or ordered probit models, respectively. Threshold parameters for these discrete variables are jointly estimated and strictly increasing.

The outcomes included in Eq. 2 depend on underlying family lifestyle in the same way as childhood adiposity and therefore include adiposity of all family members. By estimating these outcomes jointly, rather than including parental weight as independent variables in the child weight equation, we account for the endogenous effect of underlying lifestyle that is present when estimating child weight in single equation. By accounting for this endogeneity, we infer a causal effect of underlying family lifestyle on childhood adiposity.

We assume here for simplicity that there is a single latent factor but this will be tested using the exploratory factor analysis (EFA) prior to the full model being estimated. Outcomes in each period are chosen using EFA and are informed by existing literature. The outcomes of family lifestyle can differ between periods. It is assumed that there is no remaining correlation between outcomes at time *t* once the underlying factor for family lifestyle has been accounted for.

### Structural model

A structural model estimates the relationships between the latent factors; in this case, it creates the dynamic structure of underlying family lifestyle over time. This structure allows more long-term outcomes to be investigated and can show the extent to which influences can accumulate over time.

The initial underlying family lifestyle ***θ***_*i*0_, at time *t* = 0 around the time child *i* is born is

3 $$ {\boldsymbol{\theta}}_{i0}={\boldsymbol{X}}_{i0}^{\prime }{\boldsymbol{\beta}}_0+{\boldsymbol{u}}_{i0} $$

and depends on family characteristics ***X***_*i*0_, with vector of corresponding coefficients ***β***_0_. Error vector ***u***_*i*0_ is made up of two parts; the family random effect ***η***_*i*_ ~ *N*(0,*σ*_*η*_) and independent error term ${\varepsilon}_{i0}\sim N\left(0,{\sigma}_{\varepsilon_0}\right)$ which is normally, independently and identically distributed.

Similar to the stock of skills described by Heckman [58], there is a stock of family lifestyle. This stock of family lifestyle produces the observable outcomes estimated in Eqs. 1 and 2. Family lifestyle stock in one period is dependent on the stock of family lifestyle in the subsequent period of the model, so that

4 $$ {\boldsymbol{\theta}}_{it}=\alpha {\boldsymbol{\theta}}_{it-1}+{\boldsymbol{X}}_{it}^{\prime }{\boldsymbol{\beta}}_t+{\boldsymbol{u}}_{it} $$

allowing underlying family lifestyle to evolve over time following a first order autoregressive process. Independent variables ***X***_*it*_, as well as parameters *α* and ***β***_*t*_, can differ over time. Again, the error terms ***u***_*it*_ can be decomposed into a time-varying error term, ${\boldsymbol{\varepsilon}}_{it}\sim N\left(0,{\sigma}_{\varepsilon_t}\right)$ and the time-invariant unobserved family random effect, ***η***_*i*_~*N*(0, *σ*_*η*_). The inclusion of the family random effect allows us to account for any unobservable influence on underlying family lifestyle over time. This allows us to ensure that the majority of variation in the observable lifestyle outcomes are accounted for within the model.

### Model identification

One cannot identify both the means and the intercepts in Eqs. 3 and 4 because both the latent factors ***θ***_*t*_***θ***_*t*_ and the error terms are unobservable. In order to identify the model, we fix the variance of some of the error terms [51]. The variance of the error term, ***u***_0_ in Eq. 3 $\left({\sigma}_{u_0}\right)$ is fixed at 0.05 and the variance of error terms, ***u***_*t*_ in Eq. 4 $\left({\sigma}_{u_t}\right)$ is fixed at 0.01. This identifies the structural part of this model and is equivalent to restricting the variance to one (normalisation) as is done in a probit model. In this case, model convergence was more easily achieved using values smaller than one but the magnitude of these values is arbitrary. A more detailed description and proof for the identification of this model can be found in the literature [51]. The model is estimated using Mplus 6.1 [59] and data manipulation is carried out in Stata 13. More details of the estimation method are provided in Appendix 1.

### Simulations

In order to investigate the influences of underlying family lifestyle on childhood obesity, the expected means, and conditional variances of observable childhood weight status can be calculated, that is the predicted outcome of childhood weight status, conditional on other variables within the model. This equation requires the computation of several integrals and for this reason we approximate these predictions with simulations using the estimated parameters from the dynamic latent factor model. This prevents the need for the complex calculations and allows us to estimate the likelihood of obesity in children with given sets of observable characteristics and at different ages using a single model. More details on estimation using simulations are provided in Appendix 2. We use 10,000 simulated repetitions in order to stabilise the expected means. All simulations are estimated using Stata 13.

### Data

We use data from the MCS, which contains a rich set of information for a sample of 19,517 children born around the year 2000. Cohort members were recruited using child benefit records, at the time a universal benefit. The cohort members’ carers were interviewed when the child was nine months old and subsequently when they were three, five and seven years old [60]. During each of these subsequent interviews, data on height and weight were collected, amongst other adiposity measures, allowing BMI and weight status to be calculated. Ethics approval and participant consent were not necessary as this study involved the use of a previously-published de-identified database.

**Fig. 1:**
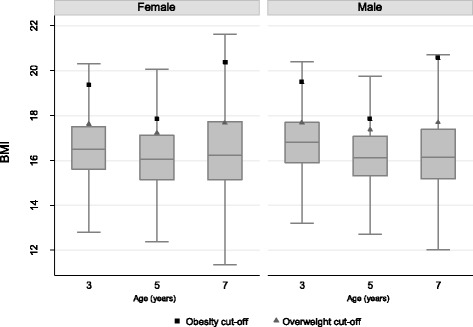
Median BMI and Interquartile Range by Age and Sex. Box plots showing median and interquartile range for BMI by age and sex using data from the Millennium Cohort Study. Outliers not included. International Obesity Taskforce (IOTF) age and sex specific cut-offs for obesity and overweight also shown

In the first wave of data, we use child weight in kilograms because weight categories are not available at nine months of age. In subsequent periods, child weight status is included using the age and sex specific International Obesity Task Force (IOTF) definitions [61], which classify children as normal weight, overweight or obese. The median and interquartile ranges of BMI by age and sex are displayed in Fig. 1 along with the IOTF cut-offs. The outcomes for the latent factors were chosen in accordance with the existing literature and using EFA [62] for each period and can be seen Table 1. These variables include maternal and paternal weight status (normal, overweight or obese), maternal smoking status (smoker, non-smoker), whether a pregnancy was planned, exclusive breastfeeding duration (never breastfed, between four and thirteen weeks, between fourteen and seventeen weeks and over seventeen weeks), screen time (3 h or more each day), regular meal times, participation in sport (never, once, twice, three times, four or more times per week), visits to the park (at least once a week), unhealthy snacking between meals and having breakfasting daily.

**Table 1:** Estimated Factor Loadings

	Factor Loading, **λ**(Eqs. 1 and 2) (Standard Error)			
Dependent Variable	Initial	Age Three Years	Age Five Years	Age Seven Years
Weight (kg)	−0.051^***^ (0.007)	–	–	–
Weight Category	–	−1.205^***^ (0.079)	−1.535^***^ (0.080)	− 1.518^***^ (0.078)
Maternal Weight Category^a^	−8.527^***^ (0.321)	−12.574^***^ (0.418)	− 12.574^***^ (0.418)	− 12.574^***^ (0.418)
Fathers Weight Category	− 1.393^***^ (0.102)	−1.215^***^ (0.088)	− 1.215^***^ (0.088)	−1.215^***^ (0.088)
Mothers’ Smoking Behaviour^b^	−0.739^***^ (0.105)	−0.757^***^ (0.101)	− 0.697^***^ (0.092)	−0.643^***^ (0.083)
Planned Pregnancy	0.712^***^ (0.079)	–	–	–
Breastfeeding Behaviour	1.056^***^ (0.064)	–	–	–
Regular Meals	–	0.577^***^ (0.091)	0.648^***^ (0.090)	–
Over Three Hours TV per day	–	−0.867^***^ (0.076)	−0.545^***^ (0.070)	− 0.431^***^ (0.062)
Sport	–	–	0.669^***^ (0.053)	0.561^***^ (0.047)
Playground/Park	–	–	0.154^***^ (0.057)	0.182^***^ (0.051)
Unhealthy Snacks	–	–	–	−0.290^***^ (0.056)
Regular Breakfast	–	–	–	0.553^***^ (0.082)
*N*	8462			

This table shows factor loadings from the factor models. ^*^p < 0.01, ^**^p < 0.05, ^***^p < 0.001, ^a^ for initial conditions this is pre-pregnancy weight category, ^b^ for initial conditions this is smoking behaviour during pregnancy

Socioeconomic and family background variables directly influence underlying lifestyle; these include variables which are found in the literature to influence the observable lifestyle outcomes outlined above. These include socioeconomic status (SES) using the five point National Statistics Socioeconomic Classification (NS-SEC) scale. The highest SES level of each of the cohort members’ parents is used to measure the cohort members’ family SES at birth. Maternal education at birth is also included. Both family SES and maternal education influence lifestyle only in the initial period of the model. This is because they do not differ a great deal over time and so any influence they have on later periods’ lifestyle is assumed to be captured through the autoregressive process. Family structure, i.e. whether the family is a two-parent or single-parent family, is included in every period of the model because it has more variation throughout early childhood. In our model, these variables all have an influence on childhood weight status through their influence on underlying family lifestyle. Ethnicity, age and sex are included as independent variables directly influencing child weight. We allow ethnicity to influence weight status in each subsequent period but because weight status is age and sex specific, age and sex are only included in the initial period.

Any observations which are not present in all four periods are removed from the analysis leaving a balanced sample of 11,484. In line with previous literature [63], children are also removed from the sample for a number of other reasons. These include children from multiple births, those weighing less than 2.5 kg at birth, those taken to a special care unit straight after birth and those whose main carer is not their natural mother. Observations are also removed from the sample when independent variables are missing. This leaves a balanced panel sample of 8462 observations.

One benefit of latent factor models is that item-non-response in the outcomes does not necessarily result in observations being removed from the analysis. A latent factor can still be estimated using the remaining outcomes, provided that there are at least two non-missing outcomes for each observation. In accordance with the World Health Organisation recommendations for biologically implausible values, childhood and parental weight statuses are recorded as missing if the height, weight or BMI values used to calculate them were implausible. Although this means that childhood adiposity outcomes were recorded as missing for some observations, this does not result in the removal of any observations.

## Results

### Model selection

Two different specifications of the dynamic latent factor model outlined above were implemented. Initially, a model was estimated with constant parameters across all periods. In this model, all lifestyle outcomes which appear in more than one period of the model had constant parameters, including factor loadings and threshold parameters. Independent variables influencing underlying family lifestyle or childhood adiposity and which appear in more than one period also had fixed parameters. In the second less restricted model, factor loadings, threshold parameters and independent variable coefficients were allowed to vary over time. All parameters were freed over time apart from the factor loadings ***λ***_*kt*_ for maternal and paternal weight categories along with their corresponding threshold parameters and the autoregressive component (*α*_*t*_). These parameter estimates are restricted over time in order to achieve convergence in the model which was not possible when they were freed. The lack of convergence is due to the large number of parameters already estimated in the model and the finite number of observations in the data. However, we also estimated models in which *α*_*t*_ was freed, but other variables were contained over time. In these models *α*_*t*_ was found to be consistent over time leading us to conclude that these are the most appropriate parameters to constraint. Restricting the autoregressive component is also in line with previous studies which restrict factors during certain stages of childhood [58].

In both the restricted and unrestricted models, the family random effect ***η***_*i*_ was found to be insignificant. This suggests that the majority of variation in the observable lifestyle outcomes is accounted for by the underlying latent factor. For this reason, and to enable the final model to converge more readily, this random effect was removed from the final models. This did not significantly affect our results.

Model fit of the unrestricted model showed an improvement on the restricted model using a likelihood ratio (LR) test as well as Akaike and Bayesian Information Criteria (AIC and BIC) supporting the claim by Cunha & Heckman (2008) that time-invariant parameters are not always best practice when analysing data on young children because they are constantly developing and changing. The remainder of this paper therefore focuses on results from the unrestricted model.

### Parameter estimates

Table 1 shows the factor loadings for each lifestyle outcome in each period of the model whilst Tables 2 and 3 present the parameter estimates relating to determinants of childhood adiposity and family lifestyle, respectively. As indicated in Table 1, all factor loadings are significant and have the expected sign; an improvement in underlying family lifestyle is associated with improved lifestyle outcomes, including but not limited to childhood adiposity. Childhood adiposity has a consistently positive and significant response to changes in the latent family lifestyle. Maternal weight status provides the largest informational content for the underlying lifestyle factor, particularly in comparison with paternal weight status, suggesting that the mother is largely responsible for the lifestyle of a family. Paternal weight status is more commonly missing than maternal weight status. However, in the majority of cases (78%), this is due to their being no father present in the household (less than 10% of the full sample). If the father is not present in the household, we assume that they do not necessarily share a common lifestyle with the rest of the family and therefore the missing data is not expected to significantly influence the results. The majority of the remainder of the missing father data is due to being unavailable at the time of interview. This could influence results if these fathers are systematically different to those who have participated. However, because this is a small proportion of observations, we do not believe it would have a large impact on results.

**Table 2:** Parameter Estimates for Covariates influencing Childhood Adiposity

	Coefficient (Eq. 2) (Standard Error)			
	Weight at first Interview (kg)	Weight Category Age 3	Weight Category Age 5	Weight Category Age 7
	**λ**			
Family Lifestyle^a^	−0.051^***^ (0.007)	−1.205^***^ (0.079)	−1.535^***^ (0.080)	− 1.518^***^ (0.078)
	**δ**			
Male	0.066^***^ (0.003)	–	–	–
Age (weeks)	0.004^***^ (0.001)	–	–	–
Black	−0.010 (0.012)	0.186 (0.113)	0.352^***^ (0.103)	0.339^***^ (0.101)
Asian	−0.077^***^ (0.007)	−0.262^***^ (0.083)	− 0.091 (0.080)	0.096 (0.076)
Other	−0.028^***^ (0.009)	−0.011 (0.092)	− 0.041 (0.097)	0.058 (0.098)
N			8462	

This table shows the parameter estimates for variables having a direct influence on childhood adiposity. ^a^ These are the factor loadings for childhood adiposity, also displayed in Table 1. ^*^p < 0.01, ^**^p < 0.05, ^***^p < 0.001

**Table 3:** Parameter Estimates for Covariates Influencing Family Lifestyle

	Coefficient (Standard Error)			
Independent Variable	Initial Family Lifestyle	Family Lifestyle Age 3	Family Lifestyle Age 5	Family Lifestyle Age 7
	α (Eq. 4)			
Previous Latent Family Lifestyle, α	–	1.094^***^ (0.007)	1.094^***^ (0.007)	1.094^***^ (0.007)
	**β**(Eqs. 3 and 4)			
Currently High SES	0.028^***^ (0.008)	–	–	–
Currently Low SES	−0.072^***^ (0.008)	–	–	–
Maternal Education at Birth	0.013^***^ (0.003)	–	–	–
Single Parent	−0.044^***^ (0.010)	−0.002 (0.007)	− 0.003 (0.005)	−0.012^**^ (0.005)

This table shows the autoregressive parameter on lifestyle and the coefficients for independent variables directly influencing underlying family lifestyle. ^*^p < 0.01, ^**^p < 0.05, ^***^p < 0.001

The proportion of variance in childhood weight status explained by underlying family lifestyle increases from 7.0% at age 3 to 11.3% by the age of 7 years, suggesting that improvements to family lifestyle could significantly reduce the likelihood of obesity in a child. This increase in the influence that lifestyle has on child weight suggests that as children get older, the influence that lifestyle has on the variation of child weight is likely to increase as children get older. Such an influence at this young age, coupled with the fact that the influence is growing, suggests that early intervention is imperative. The proportion of variance in maternal weight status explained by family lifestyle is 93.5%. This suggests that maternal weight status will be highly influenced by family lifestyle and that maternal obesity could prove useful in identifying families that need more help improving their lifestyle.

Table 2 shows the parameter estimates for variables influencing childhood adiposity in each period. It shows that boys weigh more at nine months than girls do, ceteris paribus. At nine months of age, Asian children weigh significantly less than their white counterparts do. These associations are as expected. Asian children are significantly less likely to be obese or overweight at the age of three years, but this association is insignificant by the age of five. Conversely, black children are, on average, significantly more likely than white children to be obese or overweight at the age of five and seven years.

Determinants of family lifestyle in this model are consistent with the literature. Family SES, maternal education and being from a single-parent family each have a statistically significant effect on initial latent family lifestyle. Families with high SES are at the higher end of the lifestyle distribution in the initial period and those with a low SES are towards the lower end of the distribution, ceteris paribus. Two-parent families are on average higher up the lifestyle distribution across all periods. However, this effect is only significant in the initial period and when the child is seven years old.

### Persistence of family lifestyle

From the model, we can determine the factor scores for the underlying family lifestyle factors for each individual. Factor scores are the numerical values of the underlying factors and are estimated using the observable characteristics of each observation [64]. In this case, they have no cardinal meaning but factor percentiles can be used to rank families in terms of their lifestyle to determine where each family lies on a lifestyle distribution. Families with higher factor scores have ‘healthier’ lifestyle than families with lower factor scores. We use the variance-covariance matrix from the model to calculate the proportion of variance in variables of interest explained by the latent factor and find that the variation in previous family lifestyle accounts for 98.7% of variation in current family lifestyle when the child is four years old. Table 3 shows the parameter estimates for the variables influencing these factor scores. Due to the factors having no cardinal meaning, the alpha parameter given in Table 3 can only provide the direction and significance of effect; its magnitude cannot be interpreted. Previous family lifestyle has a positive and statistically significant influence on current family lifestyle.

Correlations between the factors scores in each period are consistently above 0.982, demonstrating an immobility in the family lifestyle distribution. Table 4 shows what proportion of families remain in the same part of the lifestyle distribution over time. For example, 87.43% of families which were above the ninety-fifth percentile on the lifestyle distribution in the initial period remain above the ninety-fifth percentile when a child is seven years of age, showing some movement at the upper end of the distribution. Families that are initially in the bottom five percentiles almost never improve their lifestyle.

**Table 4:** Proportion of Families Remaining in Initial Lifestyle Percentile Group

Initial percentile	3 Years	5 Years	7 Years
≥ 95th	95.48%	91.27%	87.43%
≥ 90th	95.94%	92.77%	88.96%
≥ 75th	95.70%	93.84%	91.52%
Inter-quartile range	97.57%	96.46%	94.98%
< 25th	> 99.99%	> 99.99%	> 99.99%
< 10th	99.99%	99.99%	99.76%
< 5th	> 99.99%	> 99.99%	99.99%
N	8462		

Table 5 shows the difference in characteristics between families in the top and bottom five percentiles of the lifestyle distribution. Children in families above the 95th percentile have a lower BMI and are less likely to be obese during childhood than those from families below the 5th percentile. This differences increases as children get older as those in the lowest 5 percentiles become more likely to be obese. The most overwhelming difference between those at the upper and lower ends of this distribution is that between SES; families with low SES are almost always at the lower end of the lifestyle distribution. The information displayed in Table 5 can help to target families more likely to have unhealthy lifestyles in order to help policy makers design and target interventions more effectively and reduce inequalities in childhood obesity.

**Table 5:** Characteristics of those at Top and Bottom of Family Lifestyle Rankings

	Initial Family Lifestyle Ranking	
Variable	≥ 95th percentile	< 5th percentile
Percentage Male	49.58%	51.34%
Mean Weight (kg) (standard deviation)	8.784 (1.444)	8.935 (1.513)
Percentage Obese Age 3	4.05%	6.01%
Percentage Obese Age 5	2.06%	6.44%
Percentage Obese Age 7	2.37%	8.37%
High SES at birth	83.99%	0.42%
Low SES at birth	0.14%	98.73%
N	8462	

### Simulations

Using simulations along with the parameter estimates, it is possible to investigate a range of policy relevant relationships within this model. Here, we outline just a few which we feel are of particular policy interest. In order to investigate the inequalities in obesity prevalence between advantaged and disadvantaged children, we predict the likelihood of obesity, and the expected percentile of the lifestyle distribution, for two hypothetical children using a multidimensional measure of disadvantage. The first is an ‘advantaged’ child who is from a family with high SES, has a highly educated mother and is from a two-parent family. The second ‘disadvantaged’ child is from a family with low SES, has a poorly educated mother and is from a single-parent family. Both children are white girls and are 42.21 weeks old, the mean age of the cohort at the time of the initial MCS interviews.

Table 6 shows that the advantaged child has a lower risk of obesity than the disadvantaged child, an observation which is consistent over time. The difference is noticeable as early as the age of three years, when children from disadvantaged backgrounds are around 50% more likely to be obese than those from the most advantaged backgrounds. This difference increases with age and by the age of five years, the disadvantaged child is more than twice as likely to be obese than the advantaged child.

**Table 6:** Obesity Prevalence in Advantaged and Disadvantaged Children

	Advantaged (%)	Disadvantaged (%)
3 Years	3.79	6.43
5 Years	2.81	6.17
7 Years	2.59	6.42
N	8462	

**Fig. 2:**
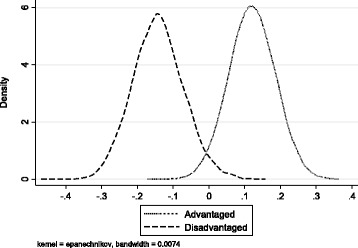
Kernel Densities of Lifestyle Distributions in Advantaged and Disadvantaged Children aged 7. Kernel density of posterior lifestyle distributions for advantaged and disadvantaged children aged seven years

Table 7 shows the expected percentile of underlying family lifestyle for the advantaged and the disadvantaged child. There is a substantial difference in the relative underlying family lifestyle between these hypothetical children from these different backgrounds. The simulated kernel density distributions of expected lifestyle for each of these hypothetical children at the age of seven years are displayed in Fig. 2 and show very little overlap in the distributions of family lifestyle between the two children. These shows how the family background characteristics, SES, maternal education and family structure, account for significant differences in underlying family lifestyle and in doing so create inequalities in childhood obesity. The model shows that much of the relationship between family background characteristics and childhood obesity can be explained by differences in lifestyle suggesting that family lifestyle mediates the relationship between family background and childhood adiposity. In addition to the parameter estimates from the dynamic latent factor model outlined above, these simulations emphasise the importance of targeting children from disadvantaged backgrounds when aiming to reduce inequalities in obesity prevalence through the use of lifestyle interventions.

**Table 7:** Lifestyle Percentiles in Advantaged and Disadvantaged Children

	Advantaged	Disadvantaged
9 months	85.63	7.50
3 Years	84.96	6.86
5 Years	84.59	6.29
7 Years	84.39	4.97
N	8462

## Discussion

This study adds to the existing literature in a number of ways. First, the latent factors used in each period allow the use of a range of outcomes to identify an underlying family lifestyle. These latent factors provide a more comprehensive measure of lifestyle compared to single-item lifestyle proxies, such as those used by many studies within the existing literature [48, 65–67]. Second, the use of latent factors also builds on previous work which used a multivariate probit model to jointly estimate a range of lifestyle behaviours but which did not directly estimate the underlying influence affecting these outcomes [46]. Third, this study uses a dynamic model of lifestyle. Previous studies investigated lifestyle variables using static or cross-sectional models [66–68]. The dynamic nature of our structural model allows the exploration of the evolution and persistence of family lifestyle during early childhood, making it possible to investigate the effects of early-life and family background influences on childhood adiposity over time. Finally, this study uses a large cohort dataset. To our knowledge, no other study has investigated the effects of underlying family lifestyle on a range of outcomes using such a large cohort. The dynamic nature of our model and the joint estimation of a range of outcomes is also important for providing economic models with more long-term evidence, which help to identify the most cost-effective interventions using fewer extrapolations and could lead to stronger public health guidance.

Our results show that improving family lifestyle could have numerous beneficial outcomes, including reducing the prevalence of childhood obesity. We find that this relationship is consistent throughout early childhood. Encouraging change in specific lifestyle behaviours cannot singlehandedly address the obesity epidemic, nor can tackling social determinants of underlying lifestyle. Simultaneously targeting the way that services interact with families to deliver health, social care and educational services to them would be the starting point to bring about change. The multiple outcomes estimated in this study, using a single dynamic model, mean that policy implications go beyond childhood obesity. The results emphasise the need for policy makers to consider the long-term influences and effects on multiple outcomes that their family lifestyle interventions could have. There are three dimensions to this. First, policy must *not* solely be about trying to bring about behaviour change. Providing advice, messages and health education about diet and lifestyle has to date been mostly ineffective especially among the most disadvantaged members of society [69, 70]. Second, policy makers and politicians must grasp the nettle that it is the life worlds of individuals, which affect the way that they live their lives and the factors that shape those life worlds must be the target for action. This means the food in and advertising industry, the pricing policies of retailers, the planning arrangements for the siting of fast food outlets for example - the elements that make up the obesogenic environment. Third, the configuration of services needs to be assessed to determine the degree to which the services currently on offer serve the need of the client groups who are supposed to benefit. If they do not serve those needs, fundamental, change is required. It will then be important to consider how these interventions might improve a number of observable outcomes for multiple family members over time as well as reducing inequalities in these outcomes. Not only is this important for policy makers but also for cost-effectiveness modellers wishing to provide robust evidence to decisions makers such as NICE on public health interventions. Current UK policies such as Change4Life have identified the need to target families rather than individuals when aiming to improve childhood outcomes. Our results provide further evidence that family interventions based on the life worlds of those families will be more successful in reducing childhood obesity interventions targeted only at the child.

We find that underlying family lifestyle is persistent and stable and is potentially part of the reason for the persistent nature of obesity over time. The persistence of family lifestyle suggests that an exogenous shock to family lifestyle, caused by an intervention or otherwise, which successfully improves underlying lifestyle, could have long-lasting influences on childhood adiposity as well as on other observable childhood outcomes and on parental adiposity, in accordance with NICE guidance [71–73]. Our findings suggest that family lifestyle interventions implemented as early in childhood as possible, will have the greatest cumulative impact on the outcomes, including childhood adiposity. Targeting the lifestyles of families with very young children or expectant parents could have effects that last throughout childhood. This is consistent with other studies which investigate early obesity interventions [74] and with studies that find that other childhood outcomes are most improved when interventions focus on the very early years [58, 75–77]. However, targeting families this early is not always possible and the persistent nature of family lifestyle suggests that successful lifestyle interventions at any stage of childhood could help to reduce obesity. The persistence of family lifestyle means that any interventions that aim to improve family lifestyle will need to be substantial in order to yield a significant improvement in family lifestyle, but that interventions successful in improving family lifestyle could produce long-lasting benefits.

Our results showed a large difference in the prevalence of childhood obesity expected in advantaged and disadvantaged children. The difference in obesity prevalence is largely explained by differences in underlying family lifestyle in advantaged and disadvantaged families. The differences in childhood obesity prevalence between advantaged and disadvantaged children increases as children get older emphasising the importance of early intervention wherever possible. We find that mobility in the family lifestyle distribution is low, particularly for those from disadvantaged backgrounds. For this reason, interventions designed to improve family lifestyle in a general population could disproportionately benefit advantaged families and are unlikely to be effective in improving the lifestyle of disadvantaged families at the more immobile lower end of the family lifestyle distribution. Therefore, in line with literature on other types of early intervention in disadvantaged children [75, 78], interventions should be designed and targeted, specifically with these disadvantaged families in mind. The reduced mobility at the unhealthier end of the family lifestyle distribution could be a result of disadvantaged families having less capability to improve their lifestyle. Therefore, improving attitudes and education relating to a healthy lifestyle would, on its own be unlikely to change the lifestyles of disadvantaged families. The National Institute for Health and Care excellence (NICE) has also recognised that disadvantage and obesity are closely related and recommends targeting specific neighbourhoods alongside more widespread childhood obesity interventions [71]. However, they do not go as far as suggesting that interventions should be specifically designed for disadvantaged families. Interventions targeted at these families will need to improve attitudes and knowledge of health lifestyles as well as improve the access to a healthy lifestyle of these families who are more likely to have budget constraints, for example, by making fresh fruit and vegetables more accessible and affordable for these families. It has been shown that disadvantaged individuals are likely to have lower self-control [79] and this should be considered when developing interventions. Improving access to healthy lifestyles in disadvantaged families is essential in helping those most in need of a positive lifestyle change and in reducing inequalities in lifestyle and therefore in childhood obesity.

We find that maternal weight is closely related to family lifestyle. Maternal weight provides the greatest informational content to the latent factor for family lifestyle in each period suggesting that maternal influences are more important when investigating family lifestyle than paternal influences. This result supports previous literature which found that maternal weight had the strongest mediating influence between SES and childhood weight when investigating a range of proxies for lifestyle [80]. This could be due to the role that mothers play as the main caregiver of young children and in the lifestyles of young families [81]. Mothers are most often responsible for family diet, exercise and other lifestyle behaviours and this could mean that underlying family lifestyle is most highly driven by maternal outcomes.

Epigenetics is one reason why maternal weight might be providing a large informational content to the family lifestyle factor. There has long been evidence of a causal relationship between health in utero and subsequent cardio vascular disease (CVD), including type 2 diabetes [82, 83]. More recently, relationships such as these have been put down to epigenetics; how shared DNA can manifest itself differently in different circumstances (for example, because of poor diet or lack of exercise), leading to children being predisposed to certain illness, including being obese [84–86] and diabetic [83]. During development in utero if a pregnant woman is subject to some external stressor, including, for example, being overweight or obese or risk factors such as lack of vitamin D or smoking during pregnancy, the developing foetus may be affected [86, 87]. This may predispose a foetus in such environments to obesity in childhood and beyond. Epigenetic transmission not only occur across a single generation, but also from grandmother to grandchild and runs through the female line [84, 87]. This could help explain why, in this study, we find that maternal weight has greater informational content for the lifestyle factor relating to weight, diet and physical activity etc. than paternal weight. Our findings suggest that any family-based lifestyle policies could be easiest implemented through maternal education and providing mothers with additional help to make it easier for them to improve the lifestyle of their family. In particular, interventions should focus on pregnant women and women of childbearing age in order to provide the best in utero environment for foetuses.

### Limitations

Although we find that the underlying factor for family lifestyle accounts for the vast majority of variation in the observable outcomes, it is possible that genetics could be playing a role here. In our sample, the mother is always the biological mother of the child but the father figure is not always a biologic father and sometimes no data on a father were collected at all. This could suggest that genetics could, to some extent, be responsible for some of the association between weight statuses of family members; child weight might be more correlated with maternal than paternal weight status due to of epigenetics. This could increase the correlation between maternal and childhood adiposity relative to the correlation between paternal and childhood adiposity, meaning that maternal weight status provides the higher informational content. We can be confident however, that any part genetics does play in this underlying factor is minimal because many of the other outcomes used to create the latent factors, are clearly related to lifestyle and not to genetics. Similarly, we found that family random effect was insignificant and therefore if we assume that genetic factors relating to obesity are constant over time, we can be confident that genetics are not having a significant influence on the relationship between family lifestyle and childhood obesity. That is not to say that genetics does not play a part in the relationship between parental and child weight status, but that the effects that we find in this study are separate from any potential effect of genetics. There is a growing literature on obesity and epigenetics and future research could further investigate the part that epigenetics plays in the effects of family lifestyle on childhood obesity.

The missing data on fathers’ weight could also influence the result that maternal weight has a much strong her influence. Missing data on paternal weight ranges from 20% in wave 1 to 36% in wave 2. However in the majority of cases, fathers who were living in the household were interviewed, a finding which is in accordance with literature on the MCS [60]. The majority of missing father data is due to their being no father present in the household. When this is the case, there could be living a very different lifestyle to the rest of the family and so should not be included in the model regardless of availability of data and would not be expected to influence the results. However, data missing for other reasons, which are not at random, is a potential limitation of this study and future research could investigate this further.

The methods used in this study assume that missing data is missing at random. Although there may be data that are missing not at random, the methods used in this study are much better than other observational methods at dealing with missing data [88]. For this reason, we do not believe that this assumption is unreasonable, given that the majority of the literature in this area makes the same assumptions but are less able to deal with missing data. In addition, studies that use the same dataset have shown that missing data caused by attrition does not significantly influence results [60, 89]. Therefore, we do not believe that missing data is a cause for concern in this study.

Our model assumes that family lifestyle has a contemporaneous influence on childhood adiposity. This could potentially be a limitation of the model given that lifestyle might take time to have an influence. For this reason, we also estimated a model in which adiposity in the subsequent period is influenced by lifestyle rather than a contemporaneous relationship. We found that the model produced similar result (due to the persistent nature of lifestyle) but had a lower likelihood than the final model presented in this study.

The MCS contains data from when a cohort child is born. However, data from before birth might have proven useful in identifying family lifestyle before the birth of a child. This would have allowed the effects of having a child on family lifestyle to be investigated. More detailed data on siblings might also have been useful and future research from later waves which contain such data could focus on the differences between individual and family effects. There could also be a cohort effect here. All children in the sample were born around the turn of the millennium; results might be slightly different for children born today. That said, given the rise in both childhood obesity and inequalities faced by disadvantaged families, any associations between the two could be even stronger.

Lifestyle within the life worlds of the family is already well established by the time a child reaches seven years old. However, as children become adolescents and increasingly interact with people outside the family home, they might be less influenced by the lifestyle of their family and could develop a more individual lifestyle as they become more independent. Further research could investigate how the dynamic path of lifestyle changes throughout childhood and into adolescence when they begin to have increasing individual influences. Likewise, further research into the intergenerational transmission of lifestyle could be useful for policy makers aiming to target families before the birth of a child.

## Conclusion

This study finds that improvements to underlying family lifestyle will have a positive influence on a range of observable lifestyle outcomes, including childhood obesity. We find that interventions should be developed at family-level rather than focussing only on the child, with a particular focus on how the mother influences the lifestyle of her family. Interventions should be implemented as early in childhood as possible, to have a larger cumulative effect and a greater chance of being successful. Successful interventions will need to be prolonged and substantial in order to overcome the persistent nature of family lifestyle. The increased immobility at the lower end of the lifestyle distribution suggests that disadvantaged families struggle to make improvements to their lifestyle despite their intentions. Interventions designed specifically for disadvantaged families as well as those targeted specifically at these families could help to reduce inequalities in childhood obesity and in lifestyle. These interventions should also consider budget constraints faced by disadvantaged families as well as improving self-control in those who want to change their behaviour but require additional support.

## Appendix 1

### Details of the Estimation Method

The dynamic latent factor model is estimated by simulated maximum likelihood using Monte Carlo integration with 3000 integration points. Robust standard errors are computed using a sandwich estimator. This requires the computation of a four-dimensional integration.

## Appendix 2

### Simulations to estimate means and conditional variances

Factor scores allow the relative standing of family lifestyle to be identified. It is the ranking of the factors scores and how easy it is for families to move up or down these rankings which provide the meaningful information. Factor scores are estimated using posterior distributions where

$$ {\boldsymbol{Y}}^{\ast }=\boldsymbol{\lambda} \vartheta +\boldsymbol{\delta} \boldsymbol{W}+\boldsymbol{\xi} $$

where ***Y***^*^ is a vector of both observed and latent responses, including the latent variable underlying child adiposity ***y****. Across all time periods, ***ϑ*** is a four-dimensional vector of latent family lifestyle factors and ***λ*** is a matrix of corresponding factor loadings. Additionally, ***W*** is a vector of independent variables with a corresponding vector of estimated coefficients ***δ***, again across all time periods, and ***ξ*** is a vector of residual errors. Additionally,

$$ \vartheta =\boldsymbol{B}\vartheta +\boldsymbol{\beta} \boldsymbol{X}+\boldsymbol{e} $$

where ***ϑ*** is a vector of the latent factor in each period, ***B*** is a four-by-four parameter matrix of the slopes for regressions of latent factor on itself at each time point, ***X*** is a vector of independent variables with corresponding coefficients, ***β***, and ***e*** = ***η*** *+* ***β*** is a vector of error terms made up of an unobserved individual random effect and residual errors. It is assumed that ***B*** has diagonal elements zero and that (***I***_4_−***B***) is non-singular.

Conditional on independent characteristics ***X*** and ***W***, the expected value of ***y*** is the mean of that conditional distribution,

$$ \left(\left.\boldsymbol{y}\right|\boldsymbol{X},\boldsymbol{W}\right)=\int \boldsymbol{y}\left[\int f\left(\left.\boldsymbol{y}\right|\vartheta, \boldsymbol{W}\right)\cdot f\left(\left.\vartheta \right|\boldsymbol{X}\right) d\vartheta \right]d\boldsymbol{y}. $$

When predicting an expected value or probability for the outcome of interest ***y***, conditional on independent variables ***X*** and ***W***, there is a conditional distribution,

$$ f\left(\left.\boldsymbol{y}\right|\boldsymbol{X},\boldsymbol{W}\right)=\int f\left(\left.\boldsymbol{y}\right|\vartheta, \boldsymbol{W}\right)\cdot f\left(\left.\vartheta \right|\boldsymbol{X}\right) d\vartheta . $$

The latent factors within the model need to be integrated out of the likelihood function in order to be estimated. This requires the computation of a four-dimensional integration. To avoid the complexities of these integrals, simulations are used to approximate them. Parameter estimates from the dynamic latent factor model can be used to simulate the likely outcomes of children and families from the sample and for those with different sets of hypothetical characteristics. The simulations which are presented in this study highlight the capabilities of this type of model to predict a range of observable outcomes.

The simulations in this study will use parameter estimates from the dynamic latent factor model estimated in Mplus 6.1 and simulations in this chapter are estimated using a user-written program in Stata 13.

## Abbreviations

AIC: Akaike information criteria
BIC: Bayesian information criteria
EFA: Exploratory factor analysis
HSE: Health Survey for England
IOTF: International obesity task force
LR: Likelihood ratio
MCS: Millennium cohort study
NICE: National Institute for Health and Care excellence
NS-SEC: National Statistics Socioeconomic Classification
SES: Socioeconomic status
